# In Vitro Evaluation of the Anticancer and Pharmacological Activities of *Eucomis comosa* (Houtt.) H.R. Wehrh.

**DOI:** 10.3390/ph19010104

**Published:** 2026-01-07

**Authors:** Thando Bhanisa, Siphamandla Qhubekani Njabuliso Lamula, Anathi Dambuza, Martha Wium, Juliano Domiraci Paccez, Luiz Fernando Zerbini, Callistus Bvenura, Lisa Valencia Buwa-Komoreng

**Affiliations:** 1 Infectious Diseases and Medicinal Plants Research Niche Area, Botany Department, Faculty of Science and Agriculture, University of Fort Hare, Private Bag X1314, Alice 5700, South Africa; 201927865@ufh.ac.za (T.B.); lbuwa@ufh.ac.za (L.V.B.-K.); 2 Department of Chemistry, Faculty of Science and Agriculture, University of Fort Hare, Private Bag X1314, Alice 5700, South Africa; 3 International Centre for Genetic Engineering and Biotechnology (ICGEB), Weirner & Beit Building, Anzio Rd, Observatory, Cape Town 7935, South Africa; mariet.wium@icgeb.org (M.W.); juliano.paccez@icgeb.org (J.D.P.); luiz.zerbini@icgeb.org (L.F.Z.); 4 Horticultural Sciences Department, Faculty of Applied Sciences, Cape Peninsula University of Technology, Symphony Way, Bellville, Cape Town 7535, South Africa; callistusb@gmail.com

**Keywords:** anticancer, *Eucomis comosa*, anti-inflammatory, DPPH, nitric oxide, FTIR, phytochemical screening

## Abstract

**Background/Objectives:** The global fight against cancer persists despite advances in prevention and treatment. The current study investigated the phytochemical constituents, antioxidant, anti-inflammatory, and anticancer properties of *Eucomis comosa*, traditionally used in South Africa to treat elephantiasis and cancer-related conditions. **Methods:** Phytochemical screening, Fourier transform infrared spectroscopy (FTIR), and liquid chromatography–mass spectrometry (LC-MS) analyses were conducted. Antioxidant activity was measured through DPPH and nitric oxide (NO) radical scavenging assays. The anticancer activity was assessed using the MTT assay. **Results:** Phytochemical screening confirmed the presence of alkaloids, cardiac glycosides, terpenoids, flavonoids, saponins, and phlobatannins. FTIR analysis of the aqueous extract displayed characteristic peaks at 3278.92 cm^−1^ for O–H stretch, at 2930.67 cm^−1^ for C–H stretch, at 1623.97 cm^−1^ for C=O stretch, 1410.24 cm^−1^ for C=C stretch and at 931.17 cm^−1^ for =C–H, while LC-MS identified diverse metabolites, including polyphenols such as flavan-3-ols, flavone glycosides, and chalcones. Among the extracts, methanol showed the strongest DPPH scavenging activity (IC_50_ = 972.73 µg/mL), followed by ethanol (1296.36 µg/mL). For NO scavenging, methanol again outperformed ethanol, with IC_50_ values of 1301 µg/mL and 2890 µg/mL, respectively. Cytotoxicity assays demonstrated that the ethanol extract completely inhibited cell growth at concentrations of 100 and 200 µg/mL. Methanol, ethanol, and hexane extracts significantly suppressed cell proliferation in DU-145, PC-3, and SKU-T-1 cancer cell lines at higher concentrations, with IC_50_ values ranging between 0.2 and 2.5 µg/mL. **Conclusions:** These findings indicate that the phytochemicals and functional groups present in *E. comosa* extracts contribute to their dose-dependent antioxidant and anticancer activities, supporting their ethnomedicinal use.

## 1. Introduction

Traditional medicinal herbs are natural plant materials utilized locally for millennia to treat illnesses, forming the bedrock of indigenous healthcare systems worldwide [1,2]. This practice remains particularly significant in Africa and other resource-limited regions, where medicinal plants remain the most accessible and affordable form of primary healthcare [3]. The World Health Organization [4] estimates that up to 80% of the population in developing countries relies on traditional medicine, predominantly plant-based remedies, for primary health care. This reliance is not only cultural but also practical, given the limited access to conventional medical services and the significant economic value of the medicinal plant trade, which is estimated to be worth billions of US dollars annually across the continent [5].

Medicinal plants owe their therapeutic potential to phytochemicals such as phenolics, terpenoids and alkaloids, which protect the plant from biotic and abiotic stress [6]. These compounds exhibit diverse pharmacological properties such as antibacterial, anticancer, and anti-inflammatory activities, making them valuable in drug discovery of new drugs [7]. Historically, plant-derived compounds have led to the development of essential drugs such as morphine, cocaine, codeine, and digitoxin which remain in clinical use today [7]. Consequently, systematic screening and characterization of medicinal plants continue to be a critical strategy for identifying novel bioactive compounds with improved pharmacological profiles.

Cancer remains a global health challenge, with a disproportionately high and growing burden in developing regions such as Africa [8,9]. Limited health care infrastructure and high treatment costs exacerbate this challenge, underscoring the urgent need for affordable and effective therapeutic options [10,11,12]. Natural products have historically contributed significantly to anticancer drug development, and the search for new plant-derived compounds with anticancer potential is ongoing [13].

*Eucomis comosa* (Asparagaceae) is a deciduous, summer-growing (December–March) bulb endemic to South Africa and growing in the Eastern Cape and Kwa-Zulu Natal provinces [14]. It thrives inland, especially in wetlands, and on grassy hillsides and is characterized by bright green leaves with purple streaks and creamy flowers with purple ovaries pollinated by insects and mammals [14]. The folkloric uses of this plant to treat ailments associated with pain and inflammation are well reported [15]. In fact, the previous authors report the use of the bulbs to treat rheumatism in the Eastern Cape and Kwa-Zulu Natal provinces. Besides old reports indicating toxicity of the plant, side effects such as rashes have been reported following administration as enemas to infants during teething, indicating widespread use of the plant [15]. Despite *E. comosa*’s ethnomedicinal relevance, scientific data on its phytochemistry and pharmacological activities remain scarce. While related species within the Eucomis genus have been studied for their therapeutic potential [16,17,18], comprehensive investigations on *E. comosa* are lacking. In fact, the only report to date indicates phytochemical studies that revealed the presence of 3-benzyl-4-chromanone and homoisoflavanones.

Therefore, the present study addresses this gap by conducting phytochemical profiling of *Eucomis comosa* and evaluating its antioxidant, anti-inflammatory, and anticancer activities, in vitro. These investigations seek to validate its folkloric uses and explore its potential as a source of novel pharmacologically active compounds.

## 2. Results

### 2.1. Phytochemical Screening

The qualitative phytochemical screening revealed the presence of alkaloids, steroids, terpenoids, flavonoids, saponins, phlobatannins, tannins, and cardiac glycosides (Table 1).

### 2.2. FTIR Spectroscopic Analysis

**Figure 1:**
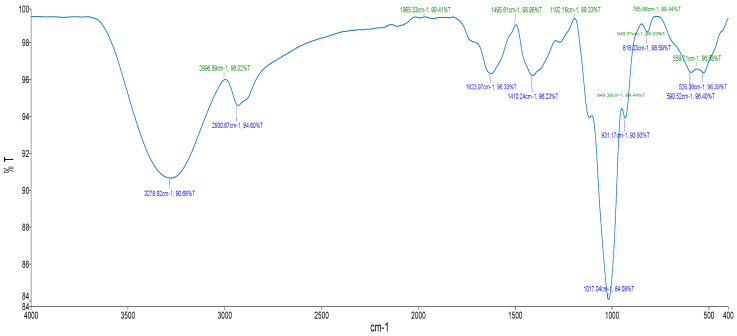
FTIR spectrum of aqueous extract from *E. comosa* (Houtt.) H.R. Wehrh.

The FTIR spectrum for the aqueous extract displayed characteristic peaks at 3278.92 cm^−1^ for O–H stretch, at 2930.67 cm^−1^ for C–H stretch, at 1623.97 cm^−1^ for C=O stretch, 1410.24 cm^−1^ for C=C stretch and at 931.17 cm^−1^ for =C–H (Figure 1 and Table 2).

### 2.3. LC-MS Screening of Secondary Metabolites

**Figure 2:**
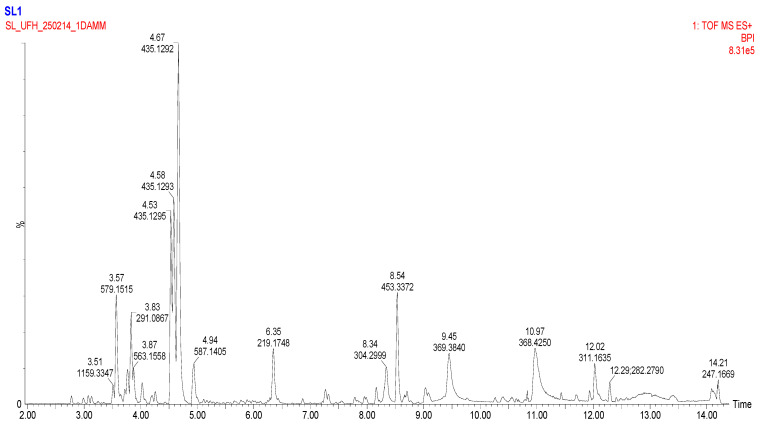
Basic Peak Chromatogram of methanolic extract of *Eucomis comosa* bulb plant; positive ion mode.

**Figure 3:**
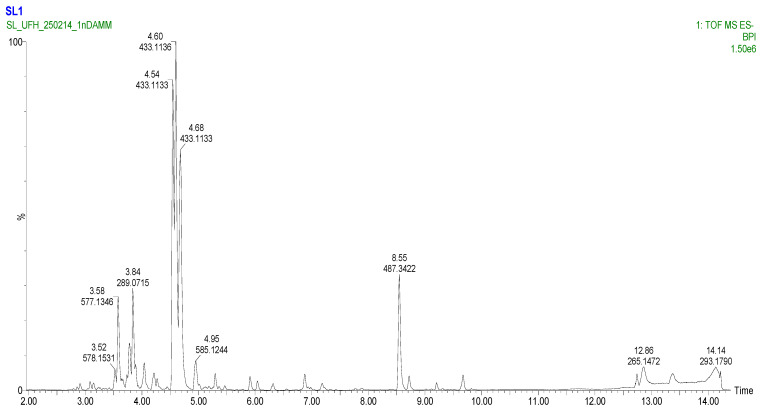
Basic Peak Chromatogram of methanolic extract of *Eucomis comosa* bulb plant; negative ion mode.

To comprehensively characterize the phytochemical composition of the methanolic extract from *Eucomis comosa* bulbs, a non-targeted metabolomic analysis was performed using UHPLC-HRMS/MS in both negative and positive electrospray ionization (ESI) modes. This dual-mode approach was critical for capturing the diverse range of metabolites present, as different chemical classes exhibit distinct ionization efficiencies. The analysis revealed a complex profile rich in specialized metabolites, predominantly polyphenols and nitrogen-containing compounds (Figure 2 and Figure 3) (Table 3 and Table 4).

The negative ion mode is highly effective for ionizing acidic compounds, resulting in the detection of a wide array of metabolites. The key identified compounds are presented in Table 3. The positive ion mode complements the analysis by efficiently ionizing alkaloids, amino acids, and certain glycosides. The key metabolites identified are listed in Table 4.

### 2.4. In Vitro Antioxidant Assays

#### 2.4.1. DPPH Radical Scavenging Assay

The concentration required to attain 50% DPPH radical scavenging effect (IC_50_) was determined from the results of a series of concentrations tested. All *E. comosa* extracts (aqueous, ethanolic, and methanolic) as well as ascorbic acid exhibited dose-dependent DPPH radical scavenging effects. The IC_50_ values were 1357.8 ± 6.3 µg/mL (aqueous), 1296.4 ± 7.2 µg/mL (ethanolic), 972.7 ± 4.5 µg/mL (methanolic), and 344.5 ± 6.6 µg/mL (ascorbic acid) (Table 5). Ascorbic acid showed the highest DPPH scavenging activity, followed by the methanolic, ethanolic, and aqueous extracts.

#### 2.4.2. Nitric Oxide Scavenging Assay

The concentration required to attain 50% NO radical scavenging effect (IC_50_) was determined from the results of a series of concentrations tested. A lower IC_50_ corresponds to a larger scavenging activity. The NO scavenging activity of *E. comosa* plant extracts (aqueous, ethanol, methanol and ascorbic acid) also revealed a dose-dependent manner of activity, similar to that of DPPH with the IC_50_ values of 2890 ± 13.7, 3563.1 ± 8.6, 1301 ± 4.3 and 723.5 ± 3.9 µg/mL, respectively (Table 5). At the tested concentrations shown in Table 5, the aqueous extract exhibited higher percentage NO inhibition at selected concentrations within the 1250–2500 µg/mL range. However, this did not correspond to the lowest IC_50_ value, indicating lower overall scavenging potency compared with the methanolic extract and ascorbic acid.

### 2.5. Anti-Inflammatory Activity

**Figure 4:**
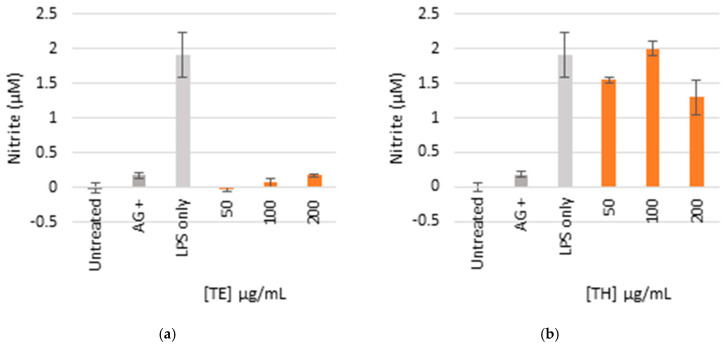

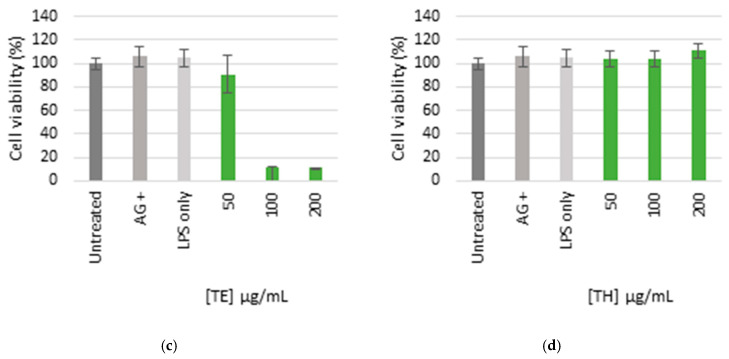
Nitric oxide production in LPS-activated macrophages (**a**,**b**) and cell viability (**c**,**d**) of cells treated with ethanol (TE) and water (TH) extracts. The bar graph represents quadruplicate values of one experiment. Error bars represent the standard deviation of the mean.

The ethanol extract (TE) was lethal to all the cells at higher concentrations between 100 and 200 µg/mL, respectively, which indicated toxicity (Figure 4). There was minimal toxicity at the lowest concentrations (50 µg/mL), while the production of NO was completely inhibited. The aqueous extract (TH) was not cytotoxic at all concentrations as the cell viability was comparable to the controls which did not indicate toxicity, and the NO was significantly inhibited at the maximum concentration of 200 µg/mL.

### 2.6. Anticancer Activity

**Figure 5:**
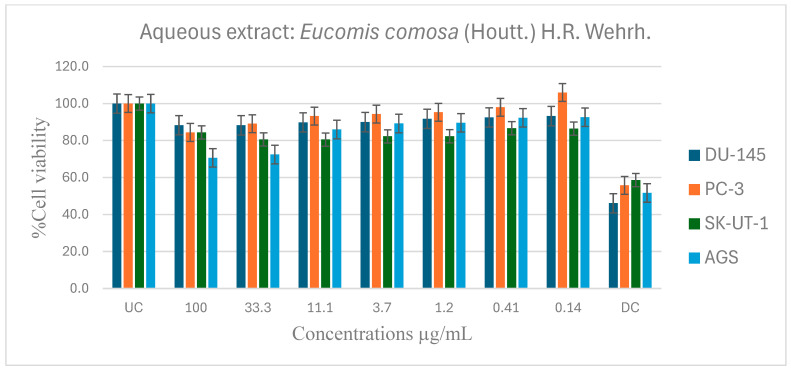
Antiproliferative effects of *E. comosa* aqueous extract against selected cancer cell lines (UC and DC mean positive control and drug control, respectively). The bars illustrate the cell viability of the different cancer cell lines at various concentrations. (UC and DC mean untreated control and drug control, respectively). Means are an average of six concentrations for each extract ± SD.

**Figure 6:**
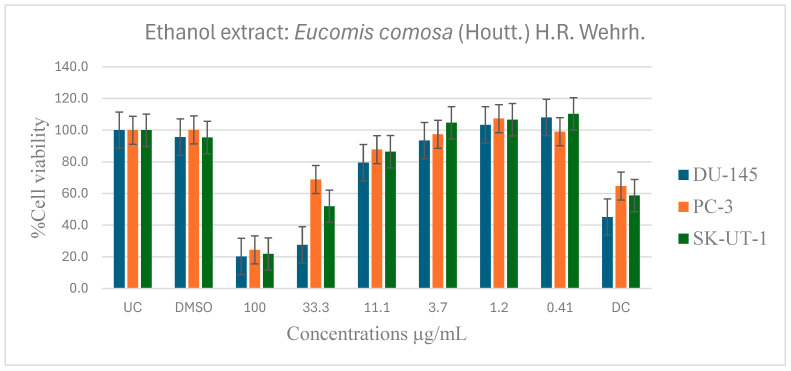
Dose-dependent antiproliferative effects of *E. comosa* ethanol extract on DU-145, PC-3, and SK-UT-1 cell lines. Cell viability is expressed as % relative to untreated control (UC) and drug control (DC). Data represent means ± SD of six concentrations per extract.

**Figure 7:**
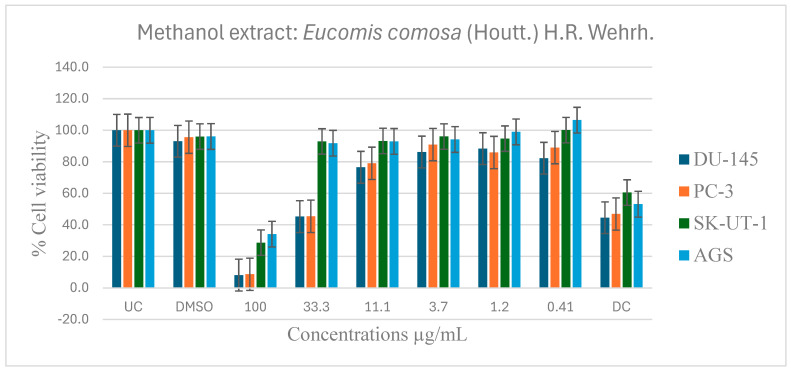
Dose-dependent antiproliferative effects of *E. comosa* methanol extract on DU-145, PC-3, SK-UT-1, and AGS cell lines. Cell viability is expressed relative to UC and DC. Data represent means ± SD of six concentrations per extract.

**Figure 8:**
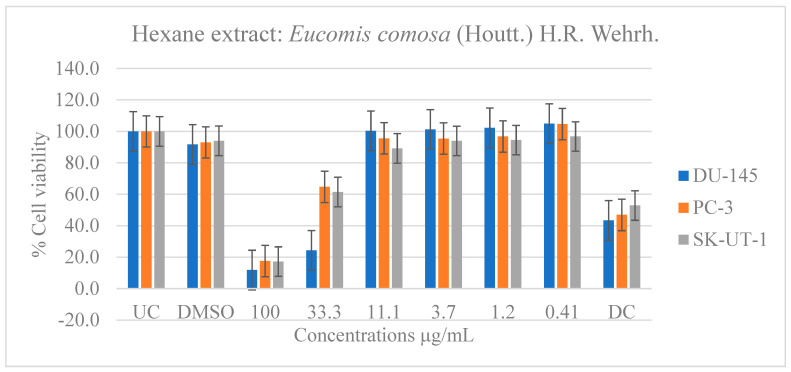
Dose-dependent antiproliferative effects of *E. comosa* hexane extract on DU-145, PC-3, and SK-UT-1 cell lines. Cell viability is expressed relative to UC and DC. Data represent means ± SD of six concentrations per extract.

Table 6 shows the results of the IC_50_ of the plant extracts on cancer cells as determined by the MTT assay. The effect of *E. comosa* aqueous, ethanol, methanol, and hexane extracts inhibition on DU-145, PC-3, SK-UT-1, and AGS cell lines are shown in Figure 5, Figure 6, Figure 7 and Figure 8. The untreated cell solution was used as a negative control, while the docetaxel (Taxotere) was used as a drug control.

When compared to the control medication, the aqueous extract showed no significant activity on any cancer cell lines, as a higher percentage cell viability was seen, indicating that it had no inhibitory effect on cancer cell growth. However, compared to the untreated control, it showed negligible dose dependency on the PC-3 cell line from 0.41 µg/mL. The DU-145 and SK-UT-1 revealed modest relevance across all concentrations, with no trending pattern and cancer cell viability ranging from 80 to 100%. In contrast, the AGS cancer cell line showed a dose-dependent pattern where cell viability decreased with increasing concentration. The maximal dosage (100 µg/mL) had the lowest cell viability (Figure 5).

Figure 6 shows the anticancer activity of the ethanol extract on the DU-145, PC-3 and SK-UT-1 cell lines. Compared to the untreated control, several significant activities were observed. The ethanol extract demonstrated significantly greater activity compared to the aqueous extract in inhibiting cell viability. The ethanol extracts inhibited cell viability of DU-145, PC-3, and SK-UT-1 at 100 µg/mL, with lC_50_ values of 2.4, 1.3, and 1.8 µg/mL, and percentages of inhibition of 79.8, 75.6, and 78.2%, respectively. The extract demonstrated a dose-dependent activity on all the cancer cell lines.

The methanol extract on DU-145 and PC-3 cell lines showed dose-dependent antiproliferative activity as the extract concentration increased. At highest doses of 100 and 33.1 µg/mL, the extract significantly inhibited cell viability relative to untreated and drug control groups. The SK-UT-1 and AGS cell lines showed modest inhibition except at the maximal concentrations of 100 µg/mL and 33.1 µg/mL, where considerable inhibition was detected, surpassing that of both the untreated and control medication (Figure 7).

The hexane extract performed similarly to the ethanol and methanol extracts and outperformed the aqueous extract. At doses of 33.3 and 100 µg/mL, the DU-145 cancer cell line showed significant inhibition of cell viability relative to the untreated and drug control groups. The PC-3 and SK-UT-1 cell lines, like the DU-145 cell line, demonstrated a greater response at a dose of 100 µg/mL compared to the drug control (Figure 8).

## 3. Discussion

### 3.1. Phytochemical Screening

The results of the current study revealed the presence of several secondary metabolites including alkaloids, steroids, terpenoids, flavonoids, saponins, phlobatannins, tannins, and cardiac glycosides. Previous studies such as that of Masondo et al. [18], revealed the presence of triterpenoid, glycosides, and homoisoflavanones from the bulbs of various *Eucomis* species. These homoisoflavanones are commonly detected in the waxy layer between the storage leaves and in the bulb tissues. The presence and diversity of flavonoids in *Eucomis* species have been linked to their pharmacological activity, particularly their anti-inflammatory potential [18]. Roy et al. [13] also identified alkaloids as an important family of bioactive molecules with validated analgesic, muscle relaxant, antioxidant, and anticancer properties. These are the foundation for medications such as atropine and vincristine. Furthermore, the discovery of cardiac glycosides in *E. comosa* is consistent with previous findings in related genera, such as *Drimia* species, which are widely valued in traditional medicine for their cardiotonic properties [19]. Due to their positive inotropic effects, glycosides play significant role in the development of pharmacotherapies for cardiovascular diseases. This association further underscores the therapeutic relevance of *E. comosa* within its familial setting.

### 3.2. Fourier Transform Infrared (FTIR) Spectroscopy Analysis

FTIR was additionally employed as a non-destructive analytical technique to identify functional groups present in the aqueous extract of *E. comosa*. This method measures the absorption of infrared radiation to generate a spectrum reflective of the sample’s chemical composition [20]. Prominent peaks were observed at 3278.92 cm^−1^ (O–H stretch), 2930.67 cm^−1^ (C–H stretch), 1623.97 cm^−1^ (C=O stretch), 1410.24 cm^−1^ (C=C stretch), and 931.17 cm^−1^ (=C–H), indicating the presence of functional groups such as alkanes, phenols, carboxyls, ketones, aromatic compounds, phosphates, alkenes, and alkyl halides. These findings are consistent with earlier investigations [20,21]. The presence of flavonoids and other phenolic compounds known for antioxidant, anticancer, antibacterial, anti-inflammatory, cardioprotective, and immune-boosting properties [22] suggests that these phytochemicals and functional groups may contribute to *E. comosa*’s therapeutic potential.

### 3.3. LC-MS Screening of Secondary Metabolites

The present study presents a comprehensive metabolomic profiling of *Eucomis comosa* bulbs using UHPLC-HRMS/MS in dual ionization modes, supported by multivariate statistical analysis (OPLS-DA) to identify key biomarkers. The resulting profile revealed a diverse chemical landscape that underpins the plant’s traditional medicinal applications. Polyphenolic compounds dominated the extract, with (-)-epicatechin and procyanidin C1 identified as major flavan-3-ols. These compounds, together with their oligomers, are reported to exhibit antioxidant and anti-inflammatory activities, potentially through mechanisms such as radical scavenging and modulation of pathways like NF-κB modulation [23]. However, it is important to note that such mechanistic effects were not directly investigated in the present study and are therefore referenced strictly as literature-based hypotheses rather than experimentally confirmed mechanisms. Nevertheless, the metabolomic profile and in vitro findings suggest that *E. comosa* may represent a potential source of natural compounds with antioxidant relevance in the context of oxidative stress–related conditions [24]. The detection of 2″-O-p-coumaroylvitexin, an acylated flavone glycoside, further strengthens this pharmacological potential. These metabolites exhibit enhanced biological activity through improved stability and uptake [25]. In addition, chalcones such as iso-salipurposide and phlorizin were identified; the latter is a well-documented sodium–glucose cotransporter (SGLT) inhibitor with established antidiabetic relevance. While the presence of these metabolites provides a plausible chemical basis for the antioxidant, anti-inflammatory, and cytotoxic activities observed in vitro, causal relationships and specific pharmacological pathways cannot be inferred without targeted functional validation [26].

Beyond polyphenols, nitrogen-containing compounds were identified, including hordenine, a phenethylamine alkaloid with reported sympathomimetic and antimicrobial properties, and a tentative steroidal glycoalkaloid, consistent with the Hyacinthaceae family’s reputation for cytotoxic, antimicrobial, and immunomodulatory metabolites [27]. These observations suggest potential pharmacological avenues beyond antioxidant activity. Notably, O-Methylsterigmatocystin, an aflatoxin precursor, was also tentatively detected [28], potentially arising from contamination by endophytic fungi [29] and plant defence metabolism [30], confirmation with analytical standards is necessary. This underscores the dual need for pharmacological exploration and safety assessment of *E. comosa* bulb preparations. In fact, toxicity concerns in this species have previously been raised but are yet to be pursed [15].

### 3.4. Antioxidant Activity

Free radicals play a crucial role in the development of chronic diseases, while plant polyphenols are important dietary components due to their antioxidant properties, which aid in the mitigation of oxidative stress-induced tissue damage. Due to the potential health concerns linked to the consumption of synthetic antioxidants, medicinal plant-derived natural alternatives are increasingly becoming popular [31]. *Eucomis* species, including *E. comosa*, are known to contain homoisoflavanones, alkaloids, saponins, and terpenoids, compounds with documented antioxidant potential [14]. In this study, the methanolic extract exhibited the highest DPPH scavenging activity, followed by ethanolic and aqueous extracts, reflecting the solvent-dependent extraction efficiency of phenolic compounds. Nitric oxide scavenging activity, however, was highest for the aqueous extract, suggesting that water-soluble bioactive compounds, potentially flavonoids and tannins, may contribute selectively to this activity [32]. These observations indicate that multiple phytochemical classes, extracted differentially by solvents, likely underpin the observed antioxidant effects. While these extracts exhibited dose-dependent antioxidant effects, the IC_50_ values were in the mg/mL range and significantly above those of typical antioxidants, suggesting moderate rather than robust antioxidant efficacy. This observation is consistent with the use of crude extracts, in which antioxidant effects likely arise from the combined and potentially synergistic action of multiple phytoconstituents present at relatively low concentrations. Collectively, these findings suggest that the antioxidant activity of *E. comosa* is biologically relevant but supportive in nature, and that further fractionation and compound-level investigations are required to identify constituents with enhanced antioxidant efficacy.

### 3.5. Anti-Inflammatory Activity

Inflammation is a physiological reaction to damaging stimuli that is characterized by vascular alterations, protein denaturation, and cellular membrane rupture. It is caused by infections, trauma, or chemical irritants and presents clinically as redness, swelling, heat, pain, and functional impairment [33]. Nitric oxide (NO), produced by inducible nitric oxide synthase (iNOS) in response to inflammatory stimuli like LPS, plays a vital role in this process [34]. In this study, *E. comosa* extracts exhibited concentration-dependent modulation of NO production. Based on preliminary screening and ethnomedicinal relevance, anti-inflammatory evaluation was limited to the aqueous and ethanolic extracts. The ethanolic extract exhibited significant suppression of NO generation at all tested concentrations (25–200 µg/mL). However, this effect coincided with significant cytotoxicity at higher concentrations (100–200 µg/mL), which limits its pharmacological relevance. This dual response suggests that NO suppression by the ethanolic extract may be partially attributable to reduced cell viability rather than selective modulation of inflammatory pathways. Although the ethanolic extract contained flavonoids, alkaloids, saponins, steroidal-related compounds and phytochemical classes previously associated with anti-inflammatory activity; however, these associations remain literature-based and were not mechanistically validated in the present study [33]. In contrast, the aqueous extract significantly inhibited NO production at 200 µg/mL without inducing cytotoxicity, indicating a comparatively safer but less potent anti-inflammatory profile. This distinction highlights a clear trade-off between efficacy and cellular tolerance, emphasizing the importance of solvent-dependent phytochemical extraction in shaping biological outcomes. Similar non-cytotoxic anti-inflammatory effects have been reported for aqueous plant extracts in LPS-activated macrophage models [35]. Although not tested on other extract types, the presence of these bioactive metabolites in ethanol and water extracts suggests a plausible link between phytochemical composition and anti-inflammatory potential. Overall, these findings underscore the exploratory nature of the anti-inflammatory activity of *E. comosa* and reinforce the need for further fractionation, selectivity assessment, and mechanistic validation to distinguish genuine anti-inflammatory effects from nonspecific cytotoxic responses.

### 3.6. Anticancer Activity

This work investigated the antiproliferative effect of *E. comosa* methanol, ethanol, hexane, and aqueous extracts on PC-3 and DU-145 (prostate cancer cell lines), uterine leiomyosarcoma (SK-UT-1), and gastric adenocarcinoma cells (AGS). The untreated cell solution, DMSO solution, and the docetaxel (Taxotere) served as controls. The plant’s phytochemicals, including phenolic acids and flavonoids, potentially contribute to its antioxidant and anti-inflammatory actions, which are important in cancer prevention [36,37,38].

In this investigation, *E. comosa* extracts showed varying cytotoxicity across cancer cell lines. The aqueous extract had modest effects on DU-145 and PC-3 prostate cancer cells but showed concentration-dependent growth suppression in (AGS) gastric cancer cells, with maximum efficacy at 1.2 µg/mL. In contrast, methanol and ethanol extracts exhibited robust cytotoxicity at greater concentrations across all examined cell lines, consistent with the findings of Jawad et al. [39], who found that polar solvents (acetone, methanol, and ethanol) exhibited more anticancer activity than aqueous extracts. The cytotoxic activity of methanolic, ethanolic, and hexane extracts against cancer cell lines suggests the contribution of multiple classes of compounds, including steroidal glycoalkaloids, alkaloids, and phenolic constituents. Hordenine and other nitrogen-containing compounds detected via LC-MS are reported to possess antimicrobial and cytotoxic effects, which may partly underline the observed anticancer activity [31]. However, the absence of parallel cytotoxicity evaluation on non-cancerous (normal) cell lines represents a significant limitation, as selectivity indices could not be determined and therapeutic relevance cannot be inferred. Consequently, the reported IC_50_ values should be interpreted strictly as indicators of general cytotoxicity rather than cancer-specific selectivity. While molecular mechanisms were not directly investigated, these metabolites provide a rational basis for the extract-dependent cytotoxicity. The presence of O-Methylsterigmatocystin, likely arising from endophytic fungi or plant metabolism, highlights the importance of future safety and toxicity assessment. Collectively, these findings show that *E. comosa* possesses some therapeutic potential, consequently necessitating more research into its processes and bioactive ingredients.

## 4. Materials and Methods

### 4.1. Plant Material Collection

The *E. comosa* plant samples were collected from the 23–30 September 2023 in Pietermaritzburg, KwaZulu-Natal Province of South Africa. Additional plant material was obtained from personnel working in nature conservation Durban market of herbalists, KwaZulu-Natal. Proper identification was completed by a taxonomist and the specimen (BUW021SBHA01) was deposited at the Griffin herbarium, Faculty of Science and Agriculture, University of Fort Hare, South Africa.

### 4.2. Preparation of Extracts

The plants were dried at ambient temperatures (24–37 °C) and ground to a fine powder. Extraction of 30 g of the dry samples in hexane, acetone, ethanol, methanol, and distilled water (arranged in increasing polarity) by shaking for 24 h followed. This was achieved on a Labcon platform shaker purchased from Laboratory Consumables, PTY, Durban, South Africa. The extracts were then filtered using Whatman No. 1 filter paper. The distilled water extract filtrate was freeze-dried (Genevac LTD, BTP-3ES00X, IP Swich, Ipswich, UK) while a rotary evaporator was used to concentrate the rest of the extracts under reduced pressure (45 °C) using a Cole Parmer SB 1100 purchased from Shanghai, China. The crude extracts were then stored at −20 °C till further use. For anticancer assays, stock solutions were prepared by dissolving 40 mg of each crude extract in 2 mL of solvent. Extracts obtained with ethanol, methanol, and hexane were solubilised in a mixture containing 0.1% DMSO, whereas the aqueous extract was dissolved in distilled water. Each extract was vortexed thoroughly and sequentially sterilized by filtration through 0.45 µm and 0.22 µm membrane filters. The resulting aliquots were wrapped in aluminum foil and stored at −20 °C until further analysis.

### 4.3. Qualitative Phytochemical Screening

Phytochemical screening of *E. comosa* was carried out using the procedures outlined by Lamula et al. [40]. Qualitative phytochemical screening was conducted on the plant material and on all solvent extracts. However, as similar phytochemical profiles were observed across extracts, the results are summarized rather than reported separately for each extract. The plant’s bulb was tested for the presence of alkaloids, tannins, saponins, anthraquinones, terpenoids, cardiac glycosides, and flavonoids. Visual observation of a change in colour or precipitate production on the addition of specified reagents determined the presence of phytochemicals.

### 4.4. LC-MS Screening for Secondary Metabolites

The bulbs were washed, freeze-dried (Christ Alpha 1-4 LDplus, Martin Christ Gefriertrocknungsanlagen GmbH, Osterode am Harz, Germany), and finely ground into a homogeneous powder using a mechanical grinder. Exactly 1.0 g of the powdered material was subjected to ultrasonication-assisted extraction (Branson 3800 Ultrasonic Cleaner, Danbury, CT, USA) with 20 mL of 80% aqueous methanol (*v*/*v*) for 30 min at room temperature. The extract was centrifuged at 10,000× *g* for 10 min (Eppendorf 5430 R, Hamburg, Germany), and the supernatant was collected. The extraction procedure was repeated twice, and the combined supernatants were filtered through a 0.22 µm PTFE syringe filter (Millipore, Burlington, MA, USA) directly into an LC-MS vial prior to analysis.

UHPLC-HRMS/MS Analysis Chromatographic separation was performed on a Vanquish Horizon UHPLC system (Thermo Fisher Scientific, Dreieich, Germany) equipped with a reversed-phase ACQUITY UPLC^®^ HSS T3 column (100 mm × 2.1 mm, 1.8 µm; Waters, Wexford, Ireland) maintained at 40 °C. The mobile phase consisted of (A) 0.1% formic acid in water and (B) 0.1% formic acid in acetonitrile. The following gradient elution programme was used: 0–2 min, 5% B; 2–15 min, 5–95% B; 15–17 min, 95% B; 17–17.1 min, 95–5% B; 17.1–20 min, 5% B for column re-equilibration. The flow rate was 0.4 mL/min, and the injection volume was 2 µL.

High-resolution mass spectrometry analysis was conducted on a Q-Exactive™ Plus Hybrid Quadrupole-Orbitrap™ Mass Spectrometer (Thermo Fisher Scientific, Germany) equipped with a Heated Electrospray Ionization (H-ESI-II) source. Data was acquired in negative ionization mode with the following parameters:

⋅ Sheath gas flow rate: 35 arb units;
⋅ Aux gas flow rate: 10 arb units;
⋅ Sweep gas flow rate: 1 arb unit;
⋅ Spray voltage: 3.2 kV;
⋅ Capillary temperature: 320 °C;
⋅ Aux gas heater temperature: 350 °C;
⋅ S-Lens RF Level: 55.0%.

The instrument was operated in a data-dependent acquisition (DDA) mode. A full MS scan (*m*/*z* 100–1500) was acquired at a resolution of 70,000 (at *m*/*z* 200) with an automatic gain control (AGC) target of 3 × 10^6^ and a maximum injection time (IT) of 100 ms. The top 5 most intense ions from the full scan were sequentially isolated (isolation window: 1.2 *m*/*z*) and fragmented via Higher-Energy C-collisional Dissociation (HCD) with normalized collision energies (NCE) stepped at 20, 40, and 60 eV. MS/MS spectra were acquired at a resolution of 17,500 with an AGC target of 1 × 10^5^ and a maximum IT of 50 ms. Dynamic exclusion was set to 15.0 s to prevent repeated fragmentation of the same abundant ions.

Data Processing and Metabolite Annotation Raw data files were processed using Compound Discoverer™ 3.3 software (Thermo Fisher Scientific). The processing workflow included:

⋅ Retention time alignment;
⋅ Peak detection (minimum peak intensity: 500,000; S/N threshold: 3);
⋅ Gap filling;
⋅ Grouping of adducts and isotopes;
⋅ Background subtraction (against a procedural blank).

Metabolite annotation was performed by querying the extracted ion features (accurate mass ± 5 µg/mL) against the following databases integrated within Compound Discoverer and online platforms: Human Metabolome Database (HMDB), mzCloud™, MassList of predicted compounds (from PubChem, ChemSpider), and the COlleCtion of Open Natural ProdUcTs (COCONUT). Annotations were validated based on three tiers of confidence, as per the Metabolomics Standards Initiative (MSI) guidelines.

### 4.5. Fourier Transform Infrared Spectroscopy Analysis

FTIR spectroscopy was employed to characterize functional groups on the plant extracts. Approximately 10 mg of the crude aqueous extract was blended with 100 mg of KBr to form a pellet, which was then mounted on a translucent disc for analysis using a Perkin Spectrum 100 FTIR spectrometer (Shelton, CT, USA). Spectral data were collected over a range of 400–4000 cm^−1^ with a resolution of 4 cm^−1^.

### 4.6. Antioxidant Assays

Antioxidant potential of the plant extracts was evaluated using two assays, viz: Nitric Oxide (NO) and 1,1-diphenyl-2-picrylhydrazyl (DPPH) radical scavenging assays. the DPPH method is widely applied for determining free radical quenching ability because the DPPH radical is relatively stable and provides a reliable measure of antioxidant activity.

#### 4.6.1. DPPH Radical Scavenging Assay

The antioxidant capacity of the plant extracts was assessed using the DPPH radical scavenging method as outlined by Dambuza et al. [41]. For the assay, 2.5 mL of a 2 mM DPPH solution in methanol was mixed with five concentrations of each extract (250, 125, 50, 10, and 5 µg/mL). Ascorbic acid served as a reference standard, and a blank was also included. The mixtures were incubated at room temperature for 30 min, after which absorbance was measured at 517 nm using a spectrophotometer. The percentage inhibition was calculated using the formula reported by Madikizela and McGaw [42].

$$\%\;DPPH\;scaveging\;activity=\frac{Absorbance\;of\;sample-Absorbance\;of\;blank}{Absornace\;of\;control-Absorbance\;of\;Blank}\times 100$$

#### 4.6.2. Nitric Oxide Scavenging Activity

Nitric oxide scavenging activity was assessed following the procedure described by Wintola and Afolayan [43]. Briefly, 2 mL of a 10 mM sodium nitroprusside solution prepared in phosphate-buffered saline (pH 7.4) was mixed with 0.5 mL of each plant extract and standard antioxidants (ascorbic acid at varying concentrations (50–500 µg/mL). The mixtures were incubated at 25 °C for 2.5 h. Subsequently, 0.1 mL of the reaction mixture was combined with 0.1 mL of Griess reagent which was prepared by dissolving 0.33% sulfanilic acid in 20% glacial acetic acid and allowed to stand for 5 min at ambient temperature. Then 1 mL of 0.1% naphthylenediamine dichloride (*w*/*v*) was added, and the samples were further incubated for 30 min at ambient temperature. Absorbance was measured at 540 nm, and all tests were performed in triplicates. The percentage inhibition of NO radicals was calculated using the equation below:

$$NO\;radical\;scaveging\;activity\left(\%\right)=\frac{Absorbance\;of\;sample-Absorbance\;of\;blank}{Absorbance\;of\;control-Absorbance\;of\;Blank}\times 100$$

### 4.7. Anti-Inflammatory Activity

The RAW 264.7 mouse macrophage cell lines were purchased from Cellonex (Johannesburg, South Africa). Sulfanilamide Solution and N-(1-Naphthyl) ethylenediamine dihydrochloride solution were made as per manufacturer’s instructions and products purchased from Promega (Madison, WI, USA). Lipopolysaccharide (LPS) and aminoguanidine were purchased from Sigma-Aldrich (St. Louise, MO, USA). RPMI1640 culture medium and fetal bovine serum (FBS) were from GE Healthcare Life Sciences (Logan, UT, USA) [44].

Sample preparation: The extracts were solubilized using dimethyl sulfoxide (DMSO) to make a stock of 100 mg/mL and stored at 4 °C until further use. Aminoguanidine (AG) was used as a positive control to indicate anti-inflammatory activity.

Anti-inflammatory screening protocol: RAW 264.7 cells were seeded in Rosswell Park Memorial Institute (RPMI1640) culture medium supplemented with 10% FBS (RPMI complete medium) into 96-well plates at a density of 1 × 10^5^ cells per well and allowed to attach overnight. The spent culture medium was removed on the following day and 50 µL sample aliquots (diluted in RPMI complete medium) added to give final concentrations of 62.5, 125 and 250 µg/mL. To assess the anti-inflammatory activity, 50 μL of LPS (final concentration of 500 µg/mL) containing medium was added to the corresponding wells. Aminoguanidine (AG) was used as a positive control at 25, 50 and 100 µg/mL. Cells were incubated for a further 24 h. To quantify NO production, 50 μL of the spent culture medium was transferred to a new 96-well plate. Sulfanilamide solution and NED solution were prepared as per manufacturer’s instructions. About 50 μL sulfanilamide solution was added to the spent culture medium and incubated for 10 min in the dark at room temperature. The 50 μL NED solution was then added to each well and further incubated for 5–10 min in the dark at room temperature.

Absorbance was measured at 540 nm (BioTek^®^ PowerWave XS spectrophotometer, Winooski, VT, USA). A standard curve using sodium nitrite dissolved in culture medium was used to determine the concentration of NO in each sample.

Cytotoxicity screening protocol: To confirm the absence of toxicity as a contributory factor, cell viability was assessed using MTT. This was performed by removing the remaining medium and treatments from each well and replacing it with fresh medium containing 0.5 mg/mL MTT, followed by incubation for 30 min at 37 °C.

Thereafter, the MTT was removed and 100 μL DMSO added to each well to solubilise the formazan crystals. The absorbance was measured at 540 nm using a BioTek^®^ PowerWave XS spectrophotometer [45].

### 4.8. Anticancer Activity

PC-3, DU-145, AGS, and SK-UT-1 cell lines were culture in Dulbecco’s Modified Eagle Medium (DMEM) (ICGEB, Cape Town, South Africa) supplemented with 10% fetal bovine serum (FBS), 1 mM L-glutamine, 100 U/mL penicillin, and 100 µg/mL streptomycin. Cultures were maintained at 37 °C in a humidified incubator with 5% CO_2_ (Thermos Fisher Scientific, Frederick, MD, USA). Cell viability was assessed using the trypan blue exclusion method [46], and cell density was determined with a haemocytometer using the formula: Cells/mL = 10^4^ × (Average count per square) × (Dilution factor) The plate was divided into the following three parts: complete media (blank), untreated cell solution as a control, and treatment with *E. comosa* extract at different concentrations. All treatments were performed in triplicates. The cytotoxic effect of the extracts was evaluated using a modified MTT assay [47]. Briefly, 6 × 10^4^ cells per well were seeded in 96-well plates with 100 µL of complete medium and allowed to adhere for 24 h. After incubation, the medium was replaced with extract solutions prepared by diluting 10 µg/mL to 200 µg/mL in complete medium. Serial dilutions were added to the wells, while untreated cells served as controls. Plates were incubated for 72 h at 37 °C in 5% CO_2_. Following treatment, 10 µL of MTT solution (2.5 µg/mL) was added to each well and incubated for 3–4 h. Formazan crystals were solubilized overnight using 10% sodium dodecyl sulfate (SDS) in 0.1 N HCl. Absorbine was measured at 595 nm using a microplate reader (Thermo Multiskan Go, Waltham, MA, USA). The mean absorbance value of the untreated control wells was taken as representing 100% cell viability. The percentage viability of untreated cells was then calculated using the following formula:

$$Percentage\;cell\;viability=\frac{Absorbance\;of\;sample}{Absorbance\;of\;control}\times 100\%$$

### 4.9. Statistical Analysis

Data visualization and sorting were performed using Microsoft Excel 2013. All experiments were conducted in triplicate. Statistical differences among treatments were evaluated using ANOVA, and significance was set up at *p* < 0.05. results are presented as mean ± standard deviation, and comparisons between means were made using Duncan’s multiple range test.

## 5. Conclusions

The pharmacological analysis of the *E. comosa* extracts revealed the presence of several phytochemicals and bioactive compounds that have been documented to possess therapeutic properties, including antiproliferation activities. Functional groups and secondary metabolites found in the plant further confirm its therapeutic potential. The results of the present study support the claim by traditional healers that *E. comosa* has medicinal and therapeutic properties; however more research needs to be performed to determine the exact compounds responsible and provide literature on this species in the *Eucomis* genus.

## Figures and Tables

**Table 1:** Qualitative phytochemical screening of *E. comosa* plant material and solvent extracts.

S/No.	Compounds	Results
**1**	Alkaloids	++
**2**	Steroids	+
**3**	Terpenoid	++
**4**	Flavonoids	++
**5**	Saponins	+
**6**	Phlobatannins	+
**7**	Tannins	++
**8**	Cardiac glycosides	+

(+): positive; A; (++): strongly positive.

**Table 2:** FTIR interpretation of functional group of aqueous extract of *E. comosa* (Houtt.) H.R. Wehrh.

Spec. No	Wavenumber (cm^−1^) (Test Samples)	Wavenumber (cm^−1^) [19]	Functional Group
**1**	3278.92	3570–3200	O–H stretch
**2**	2930.67	2935–2915	C–H stretch
**3**	1623.97	1650–1600	C=O stretch
**4**	1410.24	1600–1400	C=C stretch
**5**	1017.04	1055–1000	C–C stretch
**6**	931.17	1000–675	=C–H
**7**	590.52	730–500	C–Cl

**Table 3:** Key metabolites identified in the methanolic extract of *Eucomis comosa* bulbs using UHPLC-HRMS/MS in negative ion mode.

No.	Tentative Identification	Molecular Formula	Calculated Mass [M-H]^−^	Observed *m*/*z*	Δ (ppm)	RT (min)	Major MS/MS Fragments (*m*/*z*) ‡	Conf. Level ^1^	Relative Abundance
1	(-)-Epicatechin	C_15_H_14_O_6_	289.07176	289.07176	0.00	3.83	109.0295, 123.0452, 125.0244, 145.0295, 245.0823	1	164,885
2	2″-O-p-Coumaroylvitexin	C_30_H_26_O_12_	577.13547	577.13550	0.05	3.57	293.0455, 309.0404, 311.0561, 353.0667, 413.0876, 431.0982	2	207,652
3	Kaempferol-3-O-(6″-O-p-coumaroyl)-glucoside	C_30_H_26_O_13_	593.13038	593.13019	−0.32	3.27	285.0405, 287.0559, 409.0931, 447.0937	2	12,168
4	Procyanidin C1	C_45_H_38_O_18_	865.13419	865.13452	0.38	3.08	289.0718, 407.0776, 425.0882, 451.1036, 577.1354, 695.1491	2	16,031
5	Isosalipurposide	C_21_H_22_O_10_	433.11405	433.11407	0.05	3.77	271.0612, 285.0405, 313.0718	2	7377
6	Phlorizin	C_21_H_24_O_10_	435.12970	435.12968	−0.05	3.98	167.0347, 273.0768, 274.0846	2	4417
7	4-Hydroxybenzoic acid	C_7_H_6_O_3_	137.02442	137.02442	0.00	3.83	93.0346, 137.0244	1	8684
8	O-Methylsterigmatocystin	C_19_H_14_O_6_	337.07175	337.07148	−0.80	4.59	195.0291, 237.0408, 245.0261, 297.0639, 315.0849	2	22,498

Key: ‡ Proposed chemical formulas for indicative fragment ions are provided based on accurate mass. ^1^ Confidence Level: Level 1: Identified by comparison with an authentic standard.

**Table 4:** Key metabolites identified in the methanolic extract of *Eucomis comosa* bulbs using UHPLC-HRMS/MS in positive ion mode.

No.	Tentative Identification	Molecular Formula	Calculated Mass [M + H]^+^	Observed *m*/*z*	Δ (ppm)	RT (min)	Major MS/MS Fragments (*m*/*z*) ‡	Conf. Level ^1^	Relative Abundance
1	L-Tryptophan	C_11_H_12_N_2_O_2_	205.09771	205.09770	−0.05	2.78	118.0658, 146.0605, 188.0714	1	7780
2	Hordenine	C_10_H_15_NO	166.12264	166.12264	0.00	3.12	107.0497, 121.0653, 148.1125	2	11,625
3	Naringenin	C_15_H_12_O_5_	273.07590	273.07589	−0.04	4.58	119.0497, 147.0446, 153.0188, 177.0552	2	4991
4	Apigenin	C_15_H_10_O_5_	271.06065	271.06060	−0.18	5.21	119.0497, 153.0188, 243.0657	2	3867
5	Hexaethylene glycol	C_12_H_26_O_7_	283.17536	283.17538	0.07	3.90	205.0717, 217.0971, 247.0813, 263.0530	2	3121
6	Tentative Steroidal Glycoalkaloid	C_27_H_45_NO_8_	488.32170	488.32165	−0.10	6.54	132.0813, 414.2745, 470.3110	3	9543

Key: ‡ Proposed chemical formulas for indicative fragment ions are provided based on accurate mass. ^1^ Confidence Level: Level 1: Identified by comparison with an authentic standard.

**Table 5:** DPPH and nitric oxide (NO) scavenging activities of *Eucomis comosa* extracts.

Samples	DPPH (IC50 Value µg/mL)	NO (IC50 Value µg/mL)
AQUEOUS	1357.78 ± 6.3 ^d^	2890 ± 13.7 ^b^
ETHANOL	1296.36 ± 7.2 ^c^	3563.08 ± 8.6 ^c^
METHANOL	972.73 ± 4.5 ^b^	1301 ± 4.3 ^d^
ASCORBIC ACID (CONTROL)	344.50 ± 6.6 ^a^	723.53 ± 3.9 ^a^

Data are expressed as mean ± standard deviation. Different letters (a–d) in the same column indicate significant differences among samples (*p* < 0.05), where *n* = 3.

**Table 6:** IC_50_ values of the plant extracts on cancer cells and the percentage of inhibition as determined by the MTT assay.

Cell Lines								
Samples	DU-145	PC-3	SK-UT-1	AGS	DU-145	PC-3	SK-UT-1	AGS
Plant Extracts	IC_50_				% Inhibition at 100 µg/mL			
AQUEOUS	-	-	-	-	11.7	15.6	15.6	29.4
ETHANOL	2.4	1.3	1.8	-	79.8	75.6	78.2	-
METHANOL	2.5	2.4	0.2	0.2	91.9	91.3	71.3	65.8
HEXANE	2.3	1.6	1.7	-	88.1	82.4	82.8	-

(-): No Activity.

## Data Availability

The original contributions presented in this study are included in the article. Further inquiries can be directed to the corresponding author.
